# Metformin – a Future Therapy for Neurodegenerative Diseases: ***Theme: Drug Discovery, Development and Delivery in Alzheimer's Disease Guest Editor: Davide Brambilla***

**DOI:** 10.1007/s11095-017-2199-y

**Published:** 2017-06-06

**Authors:** Magdalena Markowicz-Piasecka, Joanna Sikora, Aleksandra Szydłowska, Agata Skupień, Elżbieta Mikiciuk-Olasik, Kristiina M. Huttunen

**Affiliations:** 1 Laboratory of Bioanalysis, Department of Pharmaceutical Chemistry Drug Analysis and Radiopharmacy, Medical University of Lodz, Muszynskiego 1, 90-151, Lodz, Poland; 2 Department of Pharmaceutical Chemistry, Drug Analysis and Radiopharmacy, Medical University of Lodz, ul., Muszyńskiego 1, 90-151, Lodz, Poland; 3 School Of Pharmacy, Faculty of Health Sciences, University of Eastern Finland,, Yliopistonranta 1C, POB 1627, 70211 Kuopio, Finland

**Keywords:** acetylcholinesterase, alzheimer’s disease, metformin, neurodegeneration, oxidative stress

## Abstract

Type 2 diabetes mellitus (T2DM) is a complex, chronic and progressive metabolic disease, which is characterized by relative insulin deficiency, insulin resistance, and high glucose levels in blood. Esteemed published articles and epidemiological data exhibit an increased risk of developing Alzheimer’s disease (AD) in diabetic pateints. Metformin is the most frequently used oral anti-diabetic drug, which apart from hypoglycaemic activity, improves serum lipid profiles, positively influences the process of haemostasis, and possesses anti-inflammatory properties. Recently, scientists have put their efforts in establishing metformin’s role in the treatment of neurodegenerative diseases, such as AD, amnestic mild cognitive impairment and Parkinson’s disease. Results of several clinical studies confirm that long term use of metformin in diabetic patients contributes to better cognitive function, compared to participants using other anti-diabetic drugs. The exact mechanism of metformin’s advantageous activity in AD is not fully understood, but scientists claim that activation of AMPK-dependent pathways in human neural stem cells might be responsible for the neuroprotective activity of metformin. Metformin was also found to markedly decease Beta-secretase 1 (BACE1) protein expression and activity in cell culture models and *in vivo*, thereby reducing BACE1 cleavage products and the production of Aβ (β-amyloid). Furthermore, there is also some evidence that metformin decreases the activity of acetylcholinesterase (AChE), which is responsible for the degradation of acetylcholine (Ach), a neurotransmitter involved in the process of learning and memory. In regard to the beneficial effects of metformin, its anti-inflammatory and anti-oxidative properties cannot be omitted. Numerous *in vitro* and *in vivo* studies have confirmed that metformin ameliorates oxidative damage.

## Introduction

Type 2 diabetes mellitus (T2DM) is a common, complex, and progressive metabolic disease, which is characterized by 3 pathophysiologic anomalies: relative insulin deficiency, insulin resistance, and hepatic insulin resistance (resulting in increased gluconeogenesis and impaired glycogen synthesis) (1,2). Importantly, the disease can lead to end-organ impairments in almost all vital organs, including the brain. According to the latest reports, cognitive decline and dementia are recognized and regarded as diabetes related complications (3,4). The relation between diabetes and cognitive impairment may be associated with metabolic lesions that occur during diabetes (such as macrovascular and microvascular changes, impaired glucose metabolism, chronic inflammation, hyperinsulinemia, insulin resistance), increased cardiovascular and life style risk factors (3–5). All these changes may lead to functional disturbances in neurons, finally resulting in cell death (6).

Lifestyle interventions supported by one anti-diabetic drug prescription initiates conventional diabetes treatment. Several drug categories have been introduced to the pharmaceutical market for the treatment of T2DM. They are as follows: sulfonylureas, meglitinides, biguanides, inhibitors of α-glucosidase, thiazolidinediones, dipeptidyl peptidase 4 inhibitors (gliptins), glucagon-like peptide-1 (GLP-1) analogues and amylin analogues (7–9). Generally, newly diagnosed patients suffering from T2DM are put on metformin monotherapy, which, in the case of insufficient glucose control, is then supplemented by other oral anti-diabetic drugs (10,11).

Metformin does not only decrease the plasma glucose level in several mechanisms, but is also characterized to beneficially effect serum lipid profiles, reduce inflammatory cell adhesion to endothelium, and exert anti-inflammatory, anti-apoptotic and anti-oxidative properties (12,13). For instance, it was found that metformin decreases interleukin 1 β (IL-1β) induced activation of proinflammatory phosphokinases Akt (protein kinase B), p38 (mitogen-activated protein kinase) (14). Taking into consideration the basic mechanisms occurring in T2DM and brain disorders, anti-diabetic dugs such as metformin might also beneficially affect the metabolism of brain cells. These effects could be regarded as clinical important for the treatment of brain complications both in T2DM and neurological diseases. On the basis of reviewing scientific literature we can imply that there is a tendency towards the application of metformin in the treatment of Alzheimer’s disease (AD) (15), amnestic mild cognitive impairment (16) and Parkinson’s disease (17). However, the presumed mechanisms underlying the neuroprotective effects of metformin in various neurodegenerative disease models are still unknown (18). Therefore, we resolved to do literature review regarding possible mechanisms connecting T2DM with AD and discuss the emerging data with particular emphasis on potential of metformin towards amelioration of neurodegeneration and its neuroprotective properties. Recent scientific papers suggest that metformin may reduce the risk of AD by its ability to sensitize neuronal insulin resistance (18,19). However, metformin was also found to increase the generation of amyloid beta (Aβ) protein (20), which, in turn, indicates that therapy with metformin may promote the development of AD (21).

On the basis of the presented results, and taking into account that T2DM increases the overall risk for the development of cognitive impairments and dementia, we decided to review the latest data on metformin application for the treatment of neurodegenerative diseases with particular emphasis on AD.

## AD, T2DM and METFROMIN

AD is the most common neurodegenerative disease defined by progressive memory shortfall and neuronal loss. The pathological characteristics of AD are as follow: extracellular amyloid plaques consisting of aggregated Aβ, intracellular neurofibrillary tangles (NFTs) comprising of hyperphosphorylated tau protein, and neuronal loss (22). Aβ develops from consecutive cleavage of the amyloid β precursor protein (APP) by β-site APP cleavage enzyme 1 (BACE1) and the γ-secretase complex (23,24). Additionally, numerous works have provided convincing evidence that AD might be regarded as a metabolic disease in which the brain becomes unable to efficiently utilize glucose for energy production and unable to respond to critical trophic factor signals due to insulin resistance (25).

The review of most recent papers and epidemiological data show an increased risk of developing AD in people with T2DM. For instance, Biessels *et al.* reported an increased risk of AD in diabetic patients (26). Kopf *et al.* also noted a heightened risk of AD in people with diabetes (27). There are also other studies indicating an elevated risk of developing AD in patients suffering from T2DM (28,29). Zhang *et al.* published an updated meta-analysis of cohort studies on the risk of AD among diabetic patients. This work included 17 studies involving 1,746,777 individuals. The results of pooling these 17 studies showed subjects with diabetes had a significantly higher incidence of AD than those without this metabolic disease (RR: 1.53, 95% CI: 1.42–1.63) (30).

Despite all the above-mentioned evidences linking diabetes and AD, there are also arguments that diabetes does not predispose to AD (31). Some clinical studies have not confirmed the association between AD and diabetes (32,33), with the exception apolipoprotein E (ApoE) ε4 allele carriers (28). ApoE, a cholesterol carrier in the brain and mediator of the uptake of lipoprotein, is coded by a gene which has three polymorphic alleles: ε2, ε3 and ε4 (34). Among them, ApoE-ε4 was identified as the strongest genetic risk factor for AD (34). Since 1996 it has been confirmed that homozygous ApoE-ε4 are responsible for increasing the risk of AD by eight folds in Caucasians (35). In addition, patients with T2DM who carry the ApoE-ε4 allele are two-fold more prone to develop AD than those without diabetes (28). Furthermore, it has been confirmed that mitochondrial damage is more severe in AD patients carrying ApoE-ε4 compared with those carrying ApoE-ε3 (36).

**Fig. 1:**
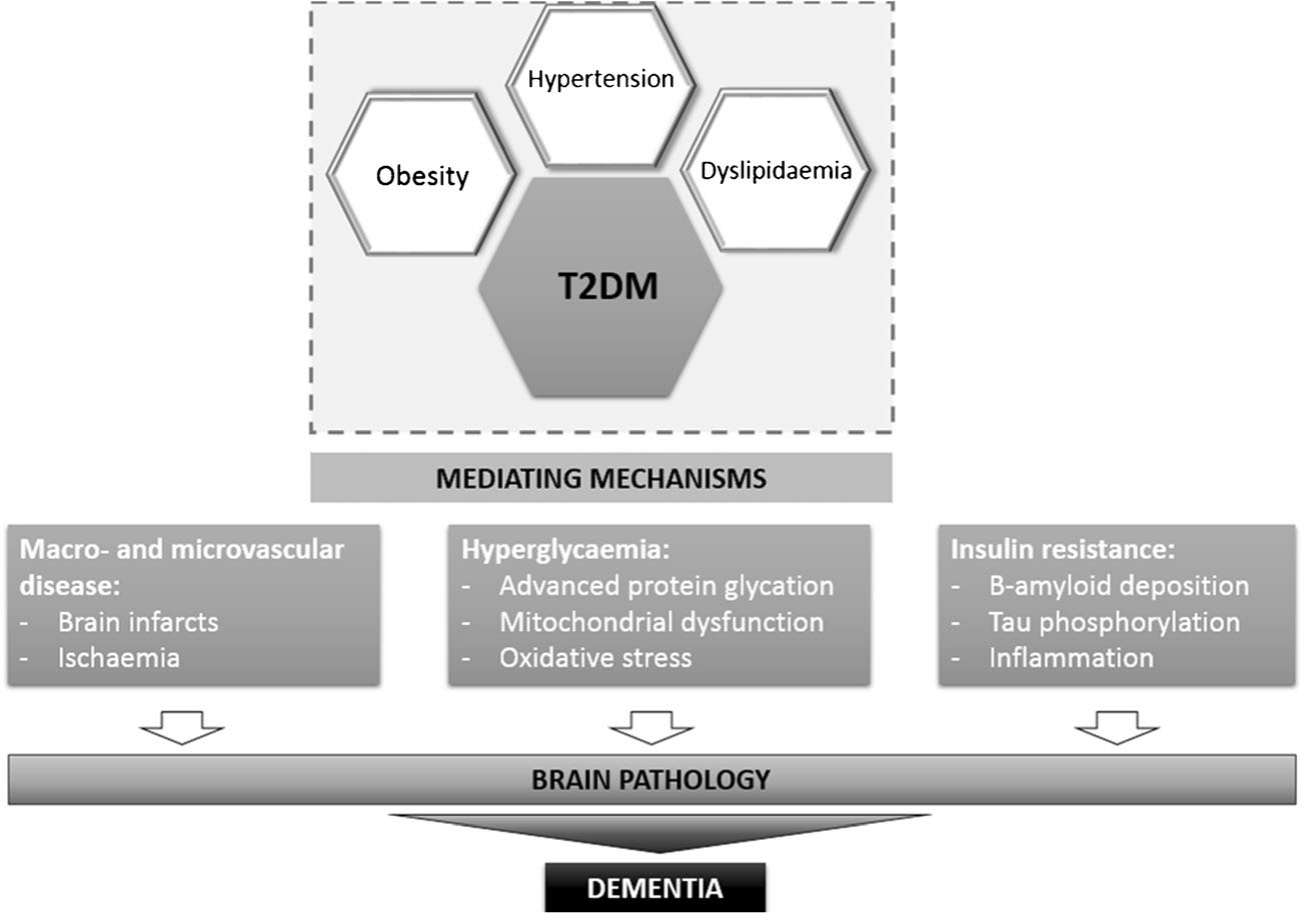
Mechanisms linking diabetes (Type 2 Diabetes Mellitus (T2DM)) and dementia. Occurrence of T2DM, as well as obesity, hypelipidaemia and hypertension are associated with and increased risk of macro- and microvascular changes, hyperglycaemia and insulin resistance giving rise to dementia (adopted from (119)).

In turn, one of the latest studies concluded that diabetes increases the risk of cerebrovascular, but not AD, pathology (31). Studies often describe increased cerebrovascular pathology (28,33). It is also worth to highlight that some studies have reported an increased risk of cerebrovascular disease (CVD) in people with T2DM (37). As far as we are concerned, the above-mentioned discrepancies may result from different definitions, types and severity of cognitive impairment, diverse ages of the study subjects, different comorbidities or lack of information about them and severity of the diabetes. Some authors claim (5) that the relationship between diabetes and cognitive decline in patients with AD has not yet been clearly established and highlight that more well-designed clinical studies are needed in order to clarify the effect of diabetes on cognitive function in patients with AD (5). Figure 1 presents a scheme summarizing the possible mechanisms linking diabetes and associated risk factors of dementia and ageing related brain changes.

Metformin, the drug which causes sensitization to insulin (ISD), apart from diabetes treatment, has also been introduced for the therapy of polycystic ovary syndrome (PCOS) (38). When describing potential beneficial effects of metformin its multidirectional influence on cardiovascular system cannot be omitted. The results of UKPDS trial showed that metformin significantly reduced (by 42%) the diabetes-related death, and also all-cause mortality (by 36%) (9,39). In one of the latest studies Mima *et al.* (40) described a clinical trial including 355 stroke patients with T2DM, and reported that administration of metformin in T2DM patients prior to stroke onset may be associated with reduced neurological severity and improved acute-phase therapy outcomes (40). The mechanisms of metformin action on cardiovascular system is not fully understood, but regarding heart muscle it has been proved that the drug promotes myocardial preconditioning, reduces cardiomyocytes apoptosis during ischemia, enhances the adaptation of cardiomyocytes metabolism during ischemia, and protects against the development of heart failure (41). Metformin was also found to prevent micro- and macro-vascular complications of diabetes mellitus by improving vascular endothelial functions (14) in AMPK-dependent way. Ladeiras-Lopes in his extensive review mentions that short-term administration of metformin ameliorates endothelial dysfunction through increased availability of NO and improved endothelium-dependent vasodilatation (42). Additionally, the results of several experimental and clinical studies highlight the multidirectional effect of metformin on haemostasis, including platelets and plasma haemostasis with both coagulation and fibrinolysis system (9). For example metformin treatment is associated with improved atherothrombotic and inflammatory blood profile such as lowered levels of plasminogen activator inhibitor type 1, TNF-α and C-reactive protein (42). One issue that might also be of importance, is the fact that metformin may exert cancer chemopreventive effects by suppressing the transformative and hyper-proliferative processes. Metformin may also improve the efficacy of various chemotherapeutics and assist in overcoming the chemotherapy resistance (43,44).

Regarding chemical structure, metformin is a biguanide (1,1-dimethylbiguanide hydrochloride) with highly hydrophilic properties which at physiological pH exists as cationic species (45,46). Bioavailability of metformin after oral administration has been estimated at approximately 50–60%, and the plasma half-life is 1.5–4 h. Unfortunately, the bioavailability shows some intra-subject, as well as inter-subject variability (47). Concerning the distribution phase of metformin it should be mentioned in this point that the drug is not bound to plasma proteins (46). Metformin diffuses into erythrocytes, in a function of time. The most important pharmacokinetic parameters are as follows: the volume of distribution (Vd) ranges from 63 to 276 L after intravenous administration, the apparent volume of distribution after oral administration (Vd/F) of 2000 mg of metformin daily is approximately 600 L (46). In the case of metabolism and excretion phase it has been established that metformin is not metabolized (48), and approximately 30–50% of an oral dose is excreted in the urine as unchanged drug within 24 h, and 30% of the dose is eliminated unchanged with the faeces (45). When describing the basic knowledge on metformin’s pharmacokinetic it should be highlighted that metformin is a substrate for several organic cation transporters (OCTs 1–3), which determine its oral absorption, distribution, elimination (hepatic uptake, renal excretion) and, importantly, biochemical effects of metformin in humans (46).

The currently available literature on metformin’s effects on the central nervous system and its potential role in AD treatment is limited and predominantly consists of *in vitro* studies and a few *in vivo* studies of short-term treatment in relatively young animals. There are also a few clinical studies attainable whose results will be discussed below.

Some studies give evidence that metformin might have beneficial effects on cognitive impairment and memory loss, while others show that metformin may be deleterious to neuronal survival. For instance, Zhao *et al.* (49) evaluated the ameliorative effects of metformin on seizures, cognitive impairment and brain oxidative stress markers observed in pentylenetetrazole-induced kindling animals. The authors confirmed that metformin suppressed the progression of kindling, ameliorated the cognitive impairment and decreased brain oxidative stress. These results led to the conclusion that metformin may be a potential preventive agent against cognitive impairment (49). Similarly, in the chronic L-methionine model of memory impairment, metformin was shown to prevent cognitive damage probably by normalizing oxidative stress in the hippocampus (50). In another study, it was shown that metformin prevents impairment of spatial reference memory associated with high fat diets in rats (51). These results were also confirmed by Ashrostaghi (52), who showed that in old rats orally administered metformin for 36 days showed a positive influence on the spatial memory performance in the Morris water maze.

According to Chen *et al.* (53), a 6 week treatment of metformin significantly ameliorates mice memory impairment, including the recovery of long-term potentiation (LTP) and normalization of several brain molecular alterations such as receptor for advanced glycation endproducts (RAGE) and nuclear factor kappa-light-chain-enhancer of activated B cells (NF-κB). In another study, McNeilly (54) investigated whether metformin treatment could diminish cognitive deficit evoked by the HF diet by improving insulin sensitivity. It was found that the drug attenuated the insulin resistance and weight gain associated with HF feeding, but had no effect on performance in operant-based matching and non-matching to position tasks (MTP/NMTP tasks) (54). Similarly, metformin treated to high fat fed mice displayed no significant effects on recognition memory in studies conducted by Lennox (55). Zhou *et al.* (56) published a study confirming metformin protective effects against cognitive deficits associated with the anti-cancer drug, cisplatin.

In the most recent study (51), Allard *et al.* examined the effect of long-term metformin administration on brain neurotrophins and cognition in aged male C57Bl/6 mice. The authors divided animals into three groups: control (C), high-fat (HF) diet and a high-fat diet supplemented with metformin (HFM). It was reported that metformin prevented impairment of spatial reference memory associated with the HF diet. The analysis of brain homogenates showed a decreased transcription of BDNF (brain-derived neurotrophic factor), NGF (nerve growth factor) and neurotrophin 3 (Nrf 3), but protein levels were not altered. Metformin was also reported to decrease expression of the antioxidant pathway regulator, Nrf2. This study reveals the need for further investigation on the long-term treatment of metformin and its potential role in altering brain biochemistry (51).

The results of some clinical studies show that long-term treatment with metformin could decrease the risk of cognitive decline (57). In addition, Ng *et al.* found no significant interactive effects of metformin use with APOE-ε4 and depression (57). Also in a study of Guo *et al.* (58), it was confirmed that a 24-week intervention with metformin improved cognitive performance in depressed patients with T2DM. Additionally, as the authors highlighted, metformin significantly improved depressive performance and changed the glucose metabolism in depressed patients with diabetes (58). Another clinical study (59) examining the effect of diabetes treatment on specific cognitive domains over 4 years, showed that only participants who used metformin alone had better cognitive function (verbal learning, working memory, and executive functions) compared to participants who used other anti-diabetic drugs (59).

On the contrary, Moore *et al.* (60) in 2013 stated that the metformin effects on cognitive performance in patients might be influenced by its dependency on vitamin B_12_ deficiency, which is widely accepted as a major cause of cognitive decline. The authors implied that vitamin B_12_ and calcium supplements may alleviate metformin-induced vitamin B_12_ deficiency (60). However, the recent study of Khattar *et al.* (61) showed that although the vitamin B_12_ levels were deficient in diabetics on metformin, this was not the reason behind the cognitive impairment.

## Potential Mechanisms of Metformin Action in AD

### The Role of AMPK Linking Metformin with β-Amyloid Formation

Chiti and Dobson (62) reported that the generation of amyloid peptides and aggregation of abnormally folded proteins are shared pathological characteristics in diabetes and AD (62). Aβ, the major protein component of amyloid plaques, can oligomerize into larger soluble assemblies, but it can also undergo conformational changes and arrange into cross-β-sheet units, forming amyloid fibrils in the senile plaques (24). Generally, it has been accepted that fibrillar Aβ are responsible for the neurotoxicity, however, a stream of research has demonstrated the significance of soluble Aβ assemblies in neuronal death (63,64).

According to Clark *et al.* (65), 90% of patients with T2DM have pancreatic islet amyloids, which are analogous to amyloid plaques in AD brains. The formation of these islet amyloids is associated with decreased β-cell mass (65). Luca *et al.* (66) showed that the molecular structure and morphology of human islet amyloid polypeptide (hIAPP) in the pancreas are similar to Aβ fibrils in AD (66). The exact functions of hIAPP are still unknown; however, studies conducted on mice models showed that the absence of hIAPP enhanced insulin secretion and improved glucose tolerance (67). Also in similar fashion, hIAPP oligomers were shown to cause β-cell death just like soluble Aβ oligomers can lead to neuronal loss (68).

A number of studies confirm that metformin has a beneficial effect on human neural stem cells (69–71). Metformin is a drug that has an evidence based impact on neuroprotection via the activation of the AMPK pathway in human neural stem cells (hNCS’s) (69). AMPK is widely known as a factor with a crucial role in the regulation of intracellular systems such as lipid metabolism, cellular glucose uptake or mitochondria biogenesis. Activation of AMPK-dependent pathways in human neural stem cells is a potential therapeutic target for AD, because cellular mechanisms of energy homeostasis are connected to AD pathogenesis (69). In studies on hNSC’s treated by Aβ, AMPK agonist (metformin) decreased cell viability and increased caspase 3/9 (a marker of caspase cascade activation) activity (69). Cells treated with Aβ exhibited that Aβ also has an influence on the induction of apoptosis by disseminate cytochrome c (a major factor in the induction of an apoptosis which activates caspase 9), which is released from the mitochondria into the cytosol. Metformin significantly prevents this release. In the same study, it was shown that metformin increases transcript levels of B-cell lymphoma 2 (Bcl-2) and cAMP Responsive Element Binding protein (CERB), targets of AMPK, thereby enhancing AMPK expression (69). AMPK is also involved in neuroprotection via signaling pathways such as Gamma Coactivator-1 α (PGC1 α), Nitrogen Response Factor 1 (NRF-1) and Transcription Factor A Mitochondrial (Tfam). The presence of Aβ inhibits this neuroprotection involvement, which leads to a decrease in both the D-loop level and mitochondrial mass. Metformin rescues PGC1 α, NRF1 and Tfam gene expression levels in hNSC. These genes were blocked by co-treatment with antagonist of AMPK (69).

The fact of the matter is that diabetes causes neurodegeneration by increased formation of advanced glycosylation end products (AGEs). AGEs are a group of molecules whose formation is initiated by a nonenzymatic reaction between sugars like glucose, and amino groups in proteins, lipids and nucleic acids (24). AGEs activate specific receptors for advanced glycation end-products (RAGE), which produce reactive oxygen species (ROS) and an inflammatory response (72). This interaction between the receptor and AGEs leads to an increase in the synthesis of the superoxide anion, which in turn reduces the activity of catalase and superoxide dismutase and simultaneously activates protein kinase C (72).

Several groups of scientists have proved that the formation and accumulation of AGEs is increased in subjects with T2DM and will determine the development of diabetic complications (73,74). In the case of AD, it has been found that AGEs colocalize with both neurofibrillary tangles and Aβ plaques, which contain three-fold higher AGEs levels in comparison with healthy controls (75). It has also been found that AGEs contribute to disease progression by accelerating the deposition of β-amyloid in different areas of the brain (76).

Accumulation of AGEs enhances hNSC cell death and mitochondrial dysfunction via downregulation of AMPK and its downstream signaling pathways. It should be mentioned here that mitochondria, and their dysfunction, have also been proposed as another fundamental link between AD and diabetes (24). In regard to diabetic patients, several abnormalities concerning mitochondria have been reported. They are as follows: increased intracellular calcium levels, deficiency in bioenergetic and antioxidant capacity, and changes in mitochondria morphology (24,77). Taking into account AD and its associations with mitochondria dysfunction, it has been found that neurons rely heavily (about 90% of the ATP required for neuron functioning) on ATP synthesis by mitochondria; therefore, they are exceptionally prone to deteriorated mitochondrial functions (78,79).

In research conducted by Chung, hNSCs were treated with AGEs and metformin. AGEs reduced cell viability and increased activity of caspase 3/9, while the treatment with metformin normalized these two factors and prevented AGEs induced cytochrome c release from the mitochondria into the cytosol in the hNSCs (70). Metformin also had an impact on RAGE, which are vital in the pathogenesis of diabetic neurodegeneration. Metformin suppressed AGE- induced upregulation of RAGE mRNA levels in hNSCs. AGEs, just like Aβ, participate in the release of cytochrome c from the mitochondria into the cytosol, which leads to apoptosis. In the mentioned studies it was shown that metformin prevents this action as well (70).

AMPK, which is activated through the phosphorylation at Thr-172, promotes diverse physiological signals that are involved in protective actions. In turn, metformin enhanced phosphorylation and weakened AGEs effects. For instance, AGEs decrease the levels of PGC1 α, NRF1 and Tfam similarly to Aβ. Metformin, by activating AMPK, provides phosphorylation of the PGC1α protein, which has the capacity to control NRF1 and Tfam transcription. Therefore, it contributes to the observed elevation in mitochondrial functions (71). The levels of ATP in AGE-treated hNSCs were lower than in vehicle controls; treatment with metformin restored ATP levels to normal. A similar situation occurred with Displacement loop (D-loop). AGEs also decreased hNSC mitochondrial mass by 60% compared to vehicle controls; treatment with metformin abrogated this effect. Maximal respiratory function, cyclooxygenase (COX) activity and mitochondrial membrane potential were decreased by exposure to AGEs, while metformin attenuated AGE induced reduction (70).

To survive, hNSCs need appropriate expression levels of peroxisome proliferator-activated receptors (PPAR- γ), Bcl-2, and CERB- genes that are targets of AMPK. Transcription of all three was significantly lowered in AGE- treated hNSC when compared to controls, but the addition of metformin reversed these effects. It should be highlighted that these effects of metformin treatment occurred in the absence of AMPK antagonist (70).

In studies conducted by DiTacchio (80), the hypothesis was that metformin treatment may provide protection against the development of AD. Scientists treated APP (β-amyloid precursor protein) mice with metformin for up to one year and tested their cognitive function by the Morris water maze test. Surprisingly, the results revealed a gender-specific response in Alzheimer’s phenotypes. Pharmacological activation of AMPK by metformin in male APP mice worsened learning and memory function. In contrast, female APP mice receiving the same treatment experienced improved learning and memory function. These findings raise the possibility that while metformin may enhance or replace part of a hormonal signaling pathway in females, it may counteract one in males (80).

In another study, Hettich *et al.* (81) claimed that metformin markedly deceased BACE1 protein expression and activity in cell culture models and *in vivo*, thereby reducing the BACE1 cleavage products and the production of Aβ (81). In turn, the next study evaluated the role of this antidiabetic drug in Aβ transport across the blood-brain barrier (BBB) (53). Chen *et al.* treated type 2 diabetic male *db/db* mice with metformin and other anti-diabetic drugs for 6 weeks and found that metformin significantly decreased the influx across the BBB via the RAGE expression and intra-arterial infusion of ^125^I–Aβ_1–40_ (53). In another *in vivo* experiment on the influence of metformin on mouse neuroblastoma cell lines, Neuro-2a (N2A) was assessed (19). The authors demonstrated that metformin markedly decreased Aβ levels under insulin resistant cell culture. Gupta and co-workers suggested that metformin ameliorates neuronal insulin resistance and AD-like changes (19). In 2012 Jiejie Li *et al.* claimed that metformin does not increase Aβ1–42 accumulation in diabetic brains (82). Scientists in their experiments on mice discovered that high levels of Aβ1–42 in the hippocampi of mice were attenuated by metformin (82).

In another study, Asadbegi *et al.* evaluated the neuroprotective influence of metformin on Aβ-induced disabilities in hippocampal synaptic plasticity in AD model rats fed a highfat (HF) (18). They treated Aβ-injected male Wistar rats with metformin and found that the drug decreased the effects of long-term potentiation (LTP) (18).

In the next study, Zhou *et al*. (83) demonstrated that pretreating rat cerebellar granule neurons (CGN) with metformin greatly enhanced cell viability against glutamate-induced neurotoxicity. Metformin significantly attenuated neuronal apoptosis in glutamate-treated CGN by reducing cytochrome c release, caspase-3 activation and phosphorylation of MAP kinases (83). In 2016 Niccoli *et al.* (84) decided to evaluate the effects of metformin on the unfolded protein response (UPR) negative master regulator expression. Authors demonstrated that genetic downregulation of Grp78 (glucose regulated protein) activity protected against Aβ toxicity which is connected with AD pathogenesis. In their *in vivo* experiments on a *Drosophila* AD model, they noticed that metformin blocked the increase in Grp78 levels correlated with Aβ expression (84).

Contrary to the above-mentioned articles, there are other studies which have shown that metformin may increase the risk of developing AD and be deleterious to neuronal survival through its AMPK activating mechanism (20,85). For instance, studies conducted on mice treated with metformin for 6 days suggested that the drug exerts an effect on APP processing via activating the AMPK pathway. Researchers found that metformin treatment in a triple transgenic mouse model of AD led to an increase in the expression of β-secretase (BACE1), one of the two enzymes that cleave APP to generate Aβ, which was accompanied by an increase in Aβ production and small plaque formation. The maximum effect of metformin was achieved in primary cultured neurons. This is a potential side-effect on accelerating AD pathogenesis and has to be taken into consideration when using metformin. In the same paper, it was also observed that metformin contributed to a decrease in Aβ levels in a transgenic mouse line in long–term therapy which lasted 3 months. In the same study they confirmed that insulin decreased accumulation of Aβ peptides (20).

Several other studies have suggested a link between chronic administration of metformin and accumulation of β-amyloid aggregates (82,86,87). For example, in 2015 Picone and co-workers (86) established that metformin increases Aβ metabolism and APP. *In vitro*, the authors explored that a higher concentration of metformin was associated with increased APP expression and consequently, the formation of Aβ fragments and aggregates. Moreover, they indicated that the drug upregulates APP and presenilin 1 gene expression in the mouse brain. Scientists have suggested that the drug exerts a similar effect *in vitro*, *ex vivo* and *in vivo* (86).

Another report looking at the effects of AMPK activation in neuronal function, (85) found that metformin application reduced late long-term potentiation in hippocampal slices, an electrophysiological correlation to memory. These studies predict that metformin treatment might be harmful to patients suffering from AD by worsening their memory function (85). One of the most recent studies showed that metformin promoted processing and aggregation of Aβ, mainly in the cortex region (87). The authors treated C57B6/J mice with metformin for seven days or three months; they noted that the drug stimulated APP processing especially in chronic administration. Futhermore, they found that metformin also increased the accumulation of Aβ aggregates in the brain cortex region. In contrast, they did not observe the presence of Aβ aggregates in the hippocampus. Scientists have confirmed that metformin directly interacts with Aβ peptide influencing its aggregation kinetics *in vitro*. According to the authors, this anti-diabetic drug induced molecular mechanisms leading to neurodegeneration in mice brains (87).

### Metformin as an AChE Inhibitor

Acetylcholinesterase (AChE) is a type of cholinesterase enzyme responsible for hydrolysis of Acetylcholine (ACh). AChE divides ACh into acetate and choline in order to avoid its accumulation, which could lead to repeated and uncontrolled muscle stimulation (88). This enzyme is homodimer and has a three-dimensional structure and ellipsoidal shape (89). The enzymic hallmark of AChE is its active-site gorge, which lengthens 20 Å into the protein and is lined with the rings of 14 highly conserved aromatic amino acids. AChE owns an active site which contains the following: (1) an esteratic site (ES) comprising of the catalytic triad Ser200-His440-Glu327, which is located at the bottom of the gorge; (2) an oxyanion hole (OAH) that stabilizes the tetrahedral intermediate binding of the carbamate carbonyl group; (3) an acyl binding site (ABS) that binds the acetyl group of ACh or the alkyl moiety of carbamate inhibitors; (4) an anionic substrate binding site (AS) that contains a small number of negative charges but many aromatic residues (90). Another typical site called the “peripheral anionic binding site” (PAS) is situated in the peripheral part of the gorge and is comprised of Trp86, Tyr337, Trp286 and Tyr72 (90).

AChE can exist in several different molecular forms that have specific patterns of expression in various cell types (91). The primary forms in the healthy adult brain are tetramers (G_4_), that are anchored in the cell membrane of neurons but also subsisting minor species, monomers (G_1_) and dimers (G_2_). In 1992 Arendt *et al.* published a paper in which they examined changes in particular species of AChE in AD; they observed a significant decrease in the G_4_ form and an incresase in the G_1_ form in the AD brain (92).

Garcia-Ayllon *et al.* (91) in their review indicated the potential participation of AChE in vicious cycles involving Aβ and P-tau. As presented by Gracia-Allon *et al.* several authors have suggested that P-tau can trigger an increase in AChE expression. In addition, it was also noticed that in the brains of a transgenic mice model with P-tau over-expression, all molecular forms of AChE, including G_4,_ were increased, contrarily to Aβ transgenic models in which only one species of AChE was increased (91).

There are several *in vivo* studies evaluating the effect of metformin on AChE activity. For instance, Bhutada *et al.* (93) tested the influence of berberine, an isoquinolone alkaloid, and metformin against cognitive dysfunction in streptozotocin-induced diabetic rats. The authors assessed lipid peroxidation and glutathione levels as parameters of oxidative stress and choline esterase (ChE) activity as a marker of cholinergic function. Induction of diabetes in rats contributed to a severe impairment in learning and memory associated with increased lipid peroxidation and ChE activity. In was found that chronic treatment (30 days) of metformin at a dose of 500 mg/kg improved cognitive performance and reduced oxidative stress and ChE activity. No statistically significant effect on ChE activity was noted in short-term administration of metformin (5 days) (93). In another study, Saliu *et al.* confirmed that metformin at a dose of 500 mg/kg significantly decreased AChE activity in the brain of streptozocin-induced diabetic rats (94). Therefore, we may presume that inhibitory effects of metformin on this key enzyme linked with neurodegeneration may be responsible in preventing cholinergic dysfunction in T2DM.

On the contrary, there are also studies which do not report anti-ChE activity of metformin. For example, Arafa *et al.* (95) examined the effect of the antidiabetic medications, canagliflozin and metformin, on the levels of cortical neurotransmitters and ChE activity in a diabetic rat model (95). The authors reported that the diabetic group exhibited a significant increase in AChE activity and a decrease in monoamine and amino acid neurotransmitter levels. Two weeks of treatment with canagliflozin led to decreased AChE activity, whereas treatment with metformin showed no significant influence on the enzyme activity (95).

In another *in vivo* experiment, the authors assessed the influence of metformin on a scopolamine-induced memory deficit model (96). Authors treated adult male Wistar rats with metformin in various doses (100 mg/kg and 300 mg/kg) and they found a dose-related effect of metformin on memory performance. The lower dose of metformin (100 mg/kg) was found to ameliorate scopolamine-induced impairments via restoring the elevated total Akt and phosphorylated tau, and enhancing phosphorylated Akt levels in the hippocampus and cortex. However, the use of metformin at both doses was not associated with the reduction of tissue AChE activity (96). The reason for such ambiguous effects in the above-mentioned studies might be the different metformin doses administered to the animals.

Apart from AChE, another enzyme, butyrylcholinesterase, was found to be a common factor in the pathogenesis of both AD and T2DM. For instance, BuChE levels were shown to be altered in T2DM patients (97), and additionally, a correlation of BuChE with insulin sensitivity has been found (98). In the case of AD, it has been found that BuChE attenuates amyloid fibril formation (99); associations of the enzyme with neurofibrillary tangles and amyloid plaques have also been found (100). According to Raygani *et al.* BuChE may modify the risk of AD alone, or in synergy with ApoE-ε 4 (101). However, no scientific reports on metformin effects on the activity or level of BuChE are available.

### Metformin as an Antioxidant

The results of many studies have proved that oxidative stress, and consequently, increased levels of its markers such as oxidised lipids and proteins, play a major role in AD pathogenesis. These changes are thought to occur in the early stages of disease development (102,103). According to Chen and Zhong the oxidation of proteins is related to diminished cerebral glucose metabolism in AD, which in turn lead to disturbances in glucose homeostasis and neuronal impairment (104). Apelt *et al.* (105) reported that an increase in reactive nitrogen species (RNS) is associated with Aβ deposition in animal model studies. The other studies have documented that Aβ may interact with mitochondrial proteins, and later on, disrupt the electron transport chain, promote mitochondria dysfunction and the generation of ROS (106). It has also been reported that oxidative stress may enhance tau hyperphosphorylation (107).

**Fig. 2:**
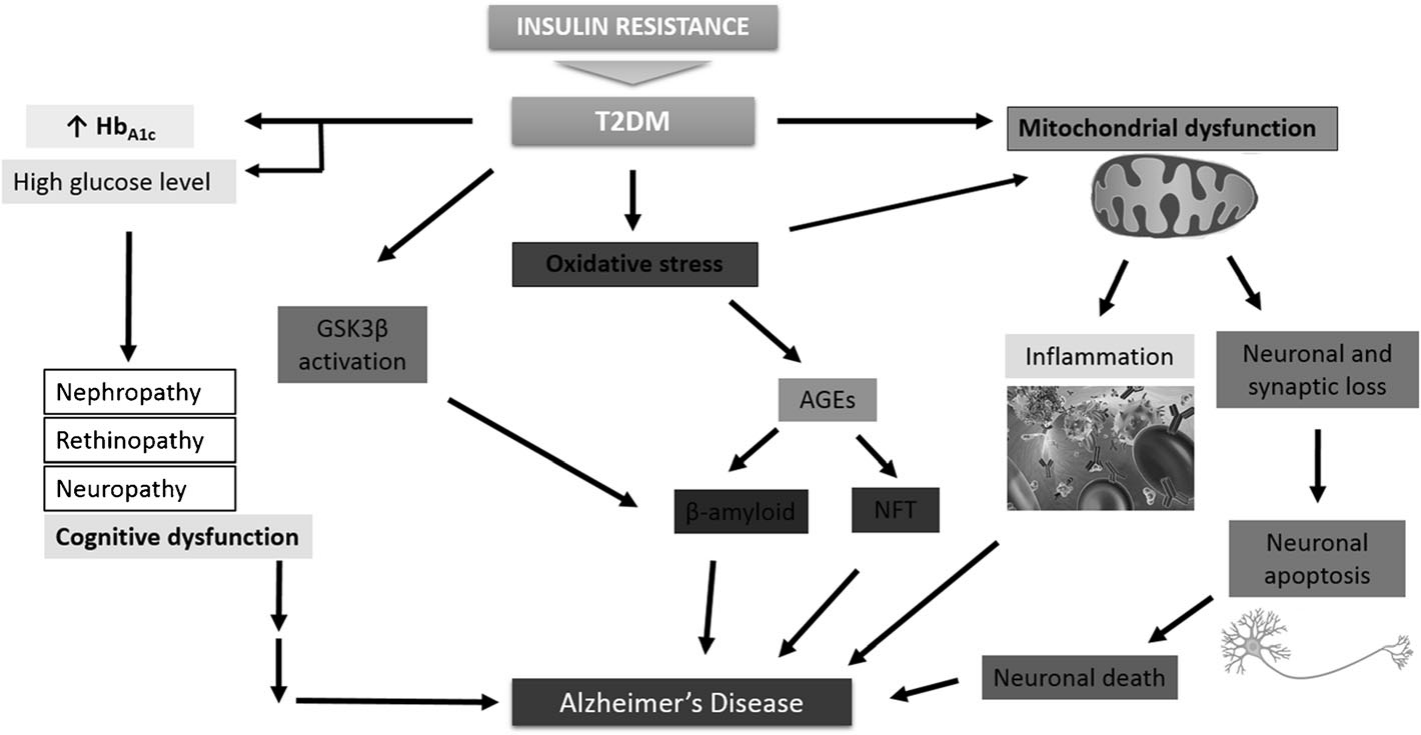
The role of insulin resistance in AD development (e.g. mitochondrial dysfunction, which contributed to neuronal death, and inflammatory process; glycosylated hemoglobin and consecutive impaired cognitive function, oxidative stress-induced β-amyloid and phosphorylated tau formations through advanced glycation end products; GSK3β - glycogen synthase kinase-3; involved in energy metabolism, promotes the hyperphosphorylation of tau (adopted from (120)).

Similar oxidative changes, such as increased levels of protein oxidation, deficits in antioxidant defences enzymes and vitamins, a lower ratio of reduced glutathione to oxidised glutathione (GSH/GSSG ratio) and increased lipid peroxidation, have also been found in diabetes patients (78,108). These processes were found to mediate diabetic neuropathy (109). It has also been found that insulin resistance occurring in AD correlates with elevated oxidative stress and DNA damage (110) (Fig. 2).

Apart from oxidative stress, inflammatory processes form a major part in the pathogenesis of T2DM, as well as AD (78). For instance, data from randomized clinical trials have shown that the development of T2DM is linked with increased levels of markers and mediators of inflammation, such as C-reactive protein (CRP) and interleukin 6 (IL-6) (111). In the case of AD, it has been proven that the degree of chronic inflammation correlates with cognitive decline (112) and brain atrophy (113). According to Bruunsgaard *et al.* (114), AD predisposes to higher levels of tumour necrosis factor α (TNF-α), which is a pro-inflammatory cytokine. Additionally, higher concentrations of TNF-α were positively correlated with the concentrations of IL-6 and CRP (114).

Studies assessing the effects of metformin treatment on oxidative stress, as well as its anti-inflammatory response, have been recently reported. For instance, in studies conducted by Ran-ran Zhao (115) it was investigated whether metformin can suppress the progression of pentylenetetrazole (PTZ)-induced oxidative stress, which develops into epileptic seizures. The studies were carried out on mice models. In the PTZ group, the level of malondialdehyde (MDA), an index of lipid peroxidation, was very high. Treatment with metformin ameliorated the oxidative damage, showing cognitive protection and anti-oxidative properties of metformin (115). In another *in vivo* study, treatment with metformin was found to reduce the expression of astrocyte and microglial markers (Glial fibrillary acidic protein (GFAP), Ionized calcium binding adaptor molecule 1 (Iba-1)) and inflammation markers (IkappaB proteins (p-IKB), interleukin 1 (IL-1) and vascular endothelial growth factor (VEGF)), while simultaneously enhancing p-AMPK and nitric oxide synthase 3 (eNOS) levels and increasing neuronal survival. These results imply the anti-inflammatory properties of metformin (116). Additionally, Dai *et al.* (117) evaluated the anti-oxidative potential of metformin in a mouse model with carbon tetrachloride (CCl_4_)-induced severe oxidative liver injuries. Treatment with metformin significantly attenuated CCl_4_-induced elevation of serum aminotransferases and hepatic histological abnormalities. Furthermore, it was found that metformin in a dose dependent manner enhanced the activity of catalase (CAT) and decreased CCl_4_-induced elevation of hepatic H_2_O_2_ levels (117). In turn, Ma *et al.* (118), in a rat model of painful diabetic neuropathy induced by streptozotocin, found that metformin decreased MDA and glycation end- products levels in blood, as well as increased superoxide dismutase activity, indicating the inhibitory effect of metformin against diabetes-induced oxidative stress. In the next animal study, Wang *et al.* (13) reported that metformin provided neuroprotection through enhancing autophagy and suppressing the inflammation and apoptosis after a spinal cord injury (SCI).

A number of studies (119–121) have proved that metformin’s pro-survival effects have been ascribed to the decreased intracellular production of ROS and improved balance between pro-oxidants and anti-oxidants. Results in the research of Khallaghi (119) demonstrated that Phosphoinositide 3-Kinase/S6 Protein Kinase (P13K/S6 K) activity is restored during metformin induced protection. Data suggests that metformin may enhance cell survival in the case of oxidative stress through strengthening anti-oxidant systems, particularly Glutathione Peroxidase (GSH) and catalase (CAT) - major detoxifying agents which degrade superoxide and H_2_O_2_ (119). In Bonnefont-Rousselot studies (120), it was revealed that metformin is able to scavenge hydroxyl free radicals, but not superoxides or hydrogen peroxides. In turn, Batchuluun *et al.* (122) evaluated the effects of metformin on production of ROS, activation of protein kinase C (PKC) and NAD(P)H oxidase, and changes to signaling molecules in response to high glucose exposure in human aortic endothelial cells. The authors found that high glucose-induced ROS overproduction was blunted by metformin. In addition, metformin demonstrated protective effects against oxidative stress by inhibiting the PKC-NAD(P)H oxidase pathway (122). In one of the most recent studies, Moon *et al.* (123) showed that metformin conferred protection against high glucose-induced apoptosis of pancreatic beta cells, mainly by interfering with ROS production and inhibiting CD36-mediated (cluster determinant 36) free fatty acid influx (123).

There are also a few clinical studies confirming metformin’s beneficial anti-oxidative properties. For example, Esteghamati (124) investigated whether the use of metformin is more effective in reducing oxidative stress compared to lifestyle modification alone. Exploration was conducted on patients diagnosed with T2DM. Advanced oxidation protein products (AOPP) and advanced glycation end products (AGEs) were measured as markers of oxidative stress. Ferritin reducing ability of plasma (FRAP) was measured to represent inherent antioxidant capacity of plasma. The results revealed that lifestyle modifications with addition of metformin treatment compared to lifestyle changes alone, caused a significant reduction in AOPP and AGE and restoration of antioxidant support assessed by FRAP. Moreover, treatment with metformin demonstrated that the drug is involved in the protection of serum lipids (especially LDL) against oxidation (124). In another clinical trial, Mirmiranpour elucidated the effects of pioglitazone on oxidative stress modulation and compared its effects with metformin (125). Newly diagnosed T2DM patients were assigned to treatment with pioglitazone (30 mg daily), metformin (1000 mg daily), or no medication. Recommendations for exercise and dietary modifications were provided in all three groups. Both anti-diabetic medications provided influential benefits on the markers of oxidative stress in patients with diabetes. The drugs effectively decreased AOPP and AGE and increased enzymatic activities of paraoxonase (PON). In addition, some differences between the drugs were found, *eg.* FRAP concentrations increased significantly with metformin. On the other hand, pioglitazone exhibited better efficacy in restoration of lecithin-cholesterol asyltransferase (LCAT) and lipoprotein lipase (LPL) enzymatic activities (125). The next clinical study aimed to evaluate the effect of metformin on different stress and inflammatory parameters in T2DM subjects (126). The authors reported that ROS generation, advanced oxidation protein products, and pentosidine were reduced by metformin treatment compared to placebo. However, it was also found that metformin enhanced total thiol and nitric oxide levels. Metformin also contributed to significant restoration of CRP. Taken together, the authors concluded that metformin therapy improves the status of oxidative and nitrosative stress altered in T2DM (126). Bułdak *et al.* (127) evaluated the impact of metformin on oxidative stress markers, antioxidative properties, inflammatory cytokines and phenotypical markers of human macrophages. The results of these experiments revealed that macrophages treated with metformin expressed less ROS, due to increased antioxidative potential. A reduction in inflammatory cytokines was also observed (127).

### Concluding Remarks and Future Prospects

Metformin, the most frequently administered anti-diabetic drug, exerts a favourable effect on body weight, lipids, and cardiovascular risk associated with T2DM. Furthermore, the review of the most recent articles indicates that metformin might also be used in the treatment of polycystic ovarian syndrome, diabetic nephropathy and metabolic syndrome. There are also some studies identifying anti-cancer and anti-ageing properties of this biguanide.

Current available evidence suggests that metformin may play an important role in the treatment of AD, as there are clinical studies confirming its beneficial effects regarding cognitive impairment and memory loss. Some authors also point out the advantageous activity of metformin on cognitive performance in depressed patients with T2DM. These favourable properties of metformin might stem from its molecular mechanism of action. For instance, the activation of AMPK by metformin has a neuroprotective effect on human neural stem cells, restores mitochondrial functions and weakens AGEs effects (70). Herein, we should also highlight the inhibitory properties of metformin on AChE, whose levels are elevated both in AD and T2DM. The results of esteemed scientific papers point out that the potential spectrum of metformin’s beneficial effects also includes anti-inflammatory and anti-oxidative properties. In various types of *in vitro* and *in vivo* studies, it has been shown that metformin ameliorates oxidative damage.

On the basis of the presented results, we can imply that metformin exerts multi-directional effects regarding AD; however, there are also some ambiguous results that make further well designed, multicentre placebo controlled and randomised clinical studies necessary.

Metfromin rapidly crosses the BBB once in circulation and distributes well into several brain regions (128). It has also been demonstrated that lipopolysaccharide (LPS)- induced inflammation in the brain does not affect metformin’s accumulation in distinct brain regions. Therefore, metformin seems to be a promising drug candidate for neurodegenerative disorders that are characterized by inflammation and oxidative stress. However, as the exact mechanisms of action of metformin are not fully understood, it is also unsure what levels of metformin are needed in which brain regions. Furthermore, the bioavailability, after oral administration, of metformin is poor (ca. 40–60%); therefore, prodrug approaches have been applied to improve its oral absorption (129–133). For example, increasing the lipophilic character of metformin and subsequently passive permeation across the cell membranes has been attempted. However, this is not a feasible approach to improve the uptake across the BBB. Instead, in the future the prodrug should be developed to utilize specific transporters expressed in the BBB, such as L-type amino acid transporter 1 (LAT1), to improve the brain uptake of metformin. In addition, the release of metformin from the lipophilic prodrugs has occurred rapidly in plasma. To reach the brain regions, the future prodrugs need to be more stable in systemic circulation and release metformin only after crossing the BBB by brain-specific enzymes.

In summary, metformin with its multi-directional properties, and safety and pharmacokinetic profile, is a promising candidate to prevent not only AD but also other neurodegenerative diseases; however, further drug development studies are worthwhile to achieve such successes.

## Abbreviations

Aβ: β-amyloid
Abs: Acyl binding site
Ach: Acetylcholine
AchE: Acetylcholinesterase
AD: Alzheimer’s disease
AGEs: Advanced glycosylation end products
Akt: Protein kinase B
AOPP: Advanced oxidation protein products
ApoE: Apolipoprotein E
APP: Amyloid β precursor protein
AS: Anionic substrate binding site
BACE: Beta-secretase 1
BBB: Blood-brain barrier
Bcl-2: B-cell lymphoma 2
BDNF: Brain-derived neurotrophic factor
BuChE: Butyrylcholinesterase
CAT: Catalase
CD: Cluster determinant
CERB: CAMP Responsive Element Binding protein
CGN: Cerebellar granule neurons
ChE: Choline esterase
COX: Cyclooxygenase
CRP: C-reactive protein
CVD: Cerebrovascular disease
D-loop: Displacement loop
eNOS: Nitric oxide synthase 3
FRAP: Ferritin reducing ability of plasma
GFAP: Glial fibrillary acidic protein
GLP-1: Glucagon-like peptide-1
Grp78: Glucose regulated protein 78
GSH: Reduced glutathione
GSSG: Oxidised glutathione
HF: High fat
HFM: High-fat diet supplemented with metformin
hNCS’s: Human neural stem cells
hIAPP: Human islet amyloid polypeptide
Iba-1: Ionized calcium binding adaptor molecule 1
IL-1: Interleukin 1
LAT1: L-type amino acid transporter 1
LCAT: Lecithin-cholesterol asyltransferase
LPL: Lipoprotein lipase
LTP: Long-term potentiation
MDA: Malondialdehyde
MTP/NMTP tasks: Matching and non-matching to position tasks
NFTs: Neurofibrillary tangles
NF-κB: Nuclear factor kappa-light-chain-enhancer of activated B cells
NGF: Nerve growth factor
NRF-1: Nitrogen Response Factor 1
Nrf 3: Neurotrophin 3
OAH: Oxyanion hole
p38: Mitogen-activated protein kinase
p-Ikb: IkappaB proteins
PAS: Peripheral anionic binding site
PGC-1 alpha: Transcriptional coactivator for steroid receptors
PKC: Protein kinase C
PON: Paraoxonase
PPAR- γ: Peroxisome proliferator-activated receptors
PTZ: Pentylenetetrazole
RAGEs: Receptor for advanced glycation endproducts
RNS: Reactive nitrogen species
ROS: Reactive oxygen species
SCI: Spinal cord injury
T2Dm: Type 2 diabetes mellitus
Tfam: Transcription Factor A Mitochondrial
TNF-α: tumour necrosis factor α
UPR: Unfolded protein response
VEGF: Vascular endothelial growth factor
